# Analysis of Copy Number Variations in Patients with Autism Using Cytogenetic and MLPA Techniques: Report of 16p13.1p13.3 and 10q26.3 Duplications

**Published:** 2016-12-05

**Authors:** Saghar Ghasemi Firouzabadi, Roshanak Vameghi, Roxana Kariminejad, Hossein Darvish, Susan Banihashemi, Mahboubeh Firouzkouhi Moghaddam, Peyman Jamali, Hassan Farbod Mofidi Tehrani, Hossein Dehghani, Mohammad Reza Raeisoon, Mehrnaz Narooie-Nejad, Javad Jamshidi, Abbas Tafakhori, Saeid Sadabadi, Farkhondeh Behjati

**Affiliations:** 1 *Genetics Research Center, University of Social Welfare and Rehabilitation Sciences, Tehran, Iran.*; 2 *Pediatric Neurorehabilitation Research Center, University of Social Welfare and Rehabilitation Sciences, Tehran, Iran.*; 3 *Kariminejad- Najmabadi Pathology and Genetics Center, Tehran, Iran.*; 4 *Department of Medical Genetics, School of Medicine, Shahid Beheshti University of Medical Sciences, Tehran, Iran.*; 5 *Child and Adolescent Psychiatry Department, Zahedan University of Medical Sciences, Zahedan, Iran.*; 6 *Research Center for Children and Adolescents Health, Zahedan University of Medical Sciences, Zahedan, Iran.*; 7 *Shahroud Welfare Organization, Shahroud, Iran.*; 8 *Health Psychology Department, Edalat University, Tehran, Iran.*; 9 *Psychiatry and Behavioral Science Research Center, Department of Social Medicine, Medicine Faculty, Birjand University of Medical Sciences, Birjand, Iran.*; 10 *Genetics of Non- Communicable Disease Research Center, Zahedan University of Medical Sciences, Zahedan, Iran.*; 11 *Non-Communicable Diseases Research Center, Fasa University of Medical Sciences, Fasa, Iran.*; 12 *Department of Neurology, School of Medicine, Imam Khomeini Hospital and Iranian Center of Neurological Research, Tehran University of Medical Sciences, Tehran, Iran.*; 13 *Bahar ducation and Rehabilitation Center for the handicapped, Tehran, Iran.*

**Keywords:** Autism, MLPA, cytogejnetic, 16p13.1p13.3 duplication, 10q26.3 duplication

## Abstract

Autism is a common neuropsychiatric disorder affecting 1 in 68 children. Copy number variations (CNVs) are known to be major contributors of autism spectrum disorder (ASD). There are different whole genome or targeted techniques to identify CNVs in the patients including karyotyping, multiplex ligation-dependent probe amplification (MLPA) and array CGH. In this study, we used karyotyping and MLPA to detect CNVs in 50 Iranian patients with autism. GTG banding and 4 different MLPA kits (2 subtelomeric and 2 autism kits) were utilized. To elevate our detection rate, we selected the sporadic patients who had additional clinical features including intellectual disability, seizure, attention deficit hyperactivity disorder, and abnormal head circumference. Two out of 50 patients (4%) showed microscopic chromosome abnormalities and 5 out of 50 (10%) demonstrated copy number gains or losses using MLPA kits. Including one overlapping result between karyotype and MLPA techniques, our overall detection rate was 6 out of 50 (12%). Three out of 6 CNVs were de novo and three others were paternally inherited. Two of CNVs detected by karyotyping and MLPA tests were 16p13.1q13.3 and 10q26.3 duplications, respectively. For these two CNVs genotype and phenotype of the patients were compared with other studies. Although the pathogenicity of cytogenetic results was certain, most of MLPA results needed to be better refined using other more accurate techniques such as array CGH. Our findings suggest that it might be possible to obtain some useful information using MLPA technique but it cannot be used as a single diagnostic tool for the autism.

Nowadays it is not needed to separate autistic disorders with different names including Asperger’s disorder, autistic disorder and pervasive developmental disorder not otherwise specified (PDD-NOS). These all have been merged together under the name of autism spectrum disorder (ASD) or the autism according to the Diagnostic and Statistical Manual of Mental Disorders, 5^th^ Edition (DSM5) criteria. ASD is a common disorder with an estimated prevalence of 1/68, characterized by persistent deficits in social relationships and communication and restricted/repetitive patterns of behavior. Many patients also have intellectual disability (70%) or epilepsy (20-25%) (1-3).

ASD is the most heritable of all neuropsychiatric disorders (4) and genetic factors play an important role in the etiology of autism. Copy number variants are known to be major contributors with the overall rate of 10-15% in children with autism from which 3-7% are cytogenetically detectable chromosome abnorma-lities (5-8) and the remaining could be identified using molecular cytogenetic techniques (9, 10). This rate increases when autism co-occurs with other clinical features, suggesting a syndromic forms of autism (11-15). Furthermore, *de novo* CNVs appear to be a more common risk factor in sporadic cases of ASD (12, 16).

Almost every chromosome has been demonstrated to be involved in imbalances contributing to autism (8, 17). Although most variants are very rare and might only be reported in a single case, several chromosomal abnormalities have been recurrently reported (5). The most frequent CNVs are duplications of maternal 15q11–q13 (18), and microdeletions of 16p11.2 (16) and *SHANK3* gene (on chromosome 22q13.3) (19, 20) occurring in 1-3%, 1% and 0.5-2% of ASD patients, respectively. The other recurrent CNVs include deletions of 7q11.23 (Williams syndrome locus), 22q11.2 (DiGeorge syndrome locus), 1q21.1 and 2q37 (5, 21, 22). Moreover, a significant proportion of cytogenetic abnormalities includes rearrangements of chromosome subtelomeric regions. Due to the gene richness of these regions, a variety of phenotypic abnormalities particularly intellectual disability (ID) plus autism are plausible to be resulted from subtelomeric rearrangements (23-25).

The most commonly used methods to detect subtelomeric and interstitial chromosomal rearrang-ements include GTG banding, fluorescence in situ hybridization (FISH), multiplex ligation-dependent probe amplification (MLPA), and microarray-based comparative genomic hybridization (array-CGH) (18). Out of these, cytogenetic is a cost-effective test that can detect all balanced and unbalanced rearrangements but with a size of at least 3-5 Mb. MLPA is a targeted but rapid and cost-effective method to screen subtelomeric abnormalities and recurrent copy number changes involved in autism (26).

In the present study, our purpose was to evaluate the diagnostic efficiency of cytogenetic and MLPA techniques in patients with autism. With regard to the finding that the highest occurrence of CNVs is demonstrated in singleton families (12, 16) and when ASD is associated with additional clinical features, we used these parameters in the present study to elevate the detection rate. Fifty sporadic patients in whom clinical diagnosis was confirmed for amtism were selected for the present study. All patients had associated features including ID, seizure, craniofacial and/ or other congenital ano-malies. GTG banding and MLPA analysis was per-formed for CNV detection. We utilized 4 different MLPA kits including 2 subtelomeric MLPA kits (P036, P070) to detect CNVs in subtelomeric region of all chromosomes, and 2 autism MLPA kits (P343 and P396) to identify imbalances in three recurrent autism loci (15q11-q13, 16p11 and *SHANK3* gene) and also in the *SHANK2*, an autism candidate gene.

## Materials and methods

**Subjects**

Clinical evaluation was carried out for the patients with ASD diagnosis. Fifty patients who met the DSM5 criteria diagnosed by pediatric neurologists specializing in autism were selected. All patients were sporadic with no family history of ASD. The selected patients had ID with one or more additional clinical features.

**GTG banding**

High resolution GTG banding technique was carried out using standard protocols. 

**MLPA**

The following 4 kits were used: SALSA P070-B2 and P036-E2 human subtelomere kits contained probes for all chromosome subtelomeric regions except the short arms of the acrocentric chromosomes. The P343- C2 AUTISM-1 probemix contained probes for three chromosomal regions re-currently involved in ASD: the 15q11-q13 (inclu-ding *UBE3A*, *GABRB3* and the 15q13 microdele-tion region with *CHRNA7*), the16p11 microdeletion region and the *SHANK3* gene at 22q13. The P396- A1 probemix contained probes for all exons of the *SHANK2* gene. MLPA analysis was performed as suggested by the manufacturer (MRC, Holland), and data analysis was carried out by Coffalyser.Net software. The MLPA test was performed twice for confirmation of abnormal changes.

**Array CGH**

Oligonucleotide- based microarray analysis was performed to determine the accurate size of two of the detected regions in two patients. The Blue Gnome Cyto Chip ISCA 8x60K v2.0 whole genome oligoarray was utilized and the tests were carried out according to the protocol provided by the manufacturer. Image analysis and base calling were performed using the BlueFuse Multi version 3 analysis software.

## Results

Two out of 50 patients demonstrated chromo-some abnormalities (4%) including one duplication on chromosome 16p and one deletion on chromo-some 15q. Furthermore, all patients were investi-gated using 4 MLPA kits (Table 1). In one patient, different results were obtained using the subtelo-meric kits (P036 and P070) that was a consequence of different genomic location of probes in two probemixes. As a result, copy number changes were demonstrated in 4 (8%) and 3(6%) patients using P036 and P070 kits, respectively. Utilizing the P343 kit, two (4%) CNVs were observed in the patients. However, none of the patients showed *SHANK2 *deletions with the P396 kit. Since some of the results were overlapping (Table 2), the total detection rate utilizing cytogenetic and MLPA analysis was 12% and MLPA showed a detection rate of 10%. Out of 6 abnormalities observed in the patients, 3 were de novo and 3 were paternally inherited. Additional clinical features presented in autism patients with copy number changes are shown in Table 3.

**Table 1:** MLPA results obtained using different MLPA kits

**Kit name**	**Detection rate**	**MLPA result**
P343	2/50	46,XX, mlpa(P343) 15q11.2q13.1x1 dn 46, XX, mlpa(P343) 15q13.3
P396	0/50	-----
P036	4/50	46, XX, mlpa 15q11.2 (P036)x1 dn 46, XY, mlpa 7qsubtel (p036) x3 pat 46, XY, mlpa 10qsubtel (p036) x3 dn 46, XY, mlpaXpsubtel (p036) x3 pat
P070	3/50	46, XX, mlpa 15q11.2 (P070)x1 46, XY, mlpa 7qsubtel (p070) x3 pat 46, XY, mlpaXpsubtel (p036) x3 pat
Total DR	4/50 (8%)	

DR: Detection rate

**Table 2:** CNVs detected using karyotype and MLPA tests

**Patient code**	**Chromosome aberration**	**Karyotyping**	**MLPA P036**	**MLPA P070**	**MLPA P343**	**MLPA P396**	**Pattern of inheritance**
p02	16p13.1p13.3 dup	+	-	-	-	-	*de novo*
p21	15q11.2 del	+	+	+	+	-	*de novo*
p27	10qsubtel dup	-	+	-	-	-	*de novo*
P32	Xpsubtel dup	-	+	+	-	-	paternal
p35	15q13 dup	-	-	-	+	-	paternal
p36	7qsubtel dup	-	+	+	-	-	paternal

**Table 3.** Additional clinical features in patients with CNVs

**Patient Code**	**Sex**	**Age (yrs)**	**ID**	**seizure**	**ADHD**	**Abnormal HC**	**Facial feature**	**Congenital anomaly**
p02	F	3	+	-	-	Microcephaly	-	Hypoplastic kidney
p21	F	11	+	+	-	Microcephaly	+	-
p27	M	17	+	+	-	Microcephaly	-	Gastrointestinal problem, immune deficiency
P32	M	13	+	-	-	-	+	Gastrointestinal problem
p35	M	7	+	+	+	Microcephaly	-	-
P36	M	12	+	-	+	-	-	-
Total abnormality (%)			6/6 (100%)	3/6 (50%)	2/6 (33%)	4/6 (67%)	2/6 (33%)	3/6 (50%)

**Cytogenetic abnormalities**

**Fig. 1:**
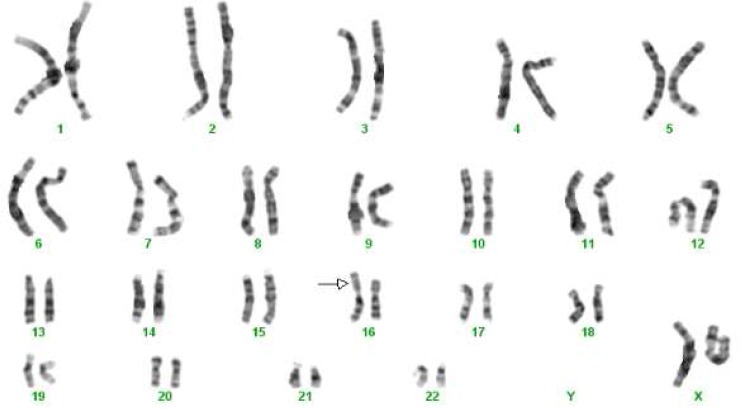
The patient (p2) karyotype: 46,XX, add (16)(p13.1) de novo

Patient one (p2) was a 3-year-old girl with autism, ID, microcephaly, hypoplastic right kidney and minor facial feature including low set hairline and broad/depressed nasal bridge. In cytogenetic study she showed a large duplication on chromosome 16p. Her karyotype was 46, XX, add(16)(p13.1) (Figure 1) and her parents had normal karyotypes. MLPA with subtelomeric and autism P343 kits containing probes for the 16p subtelomeric and 16p11.2 regions demonstrated normal results. The duplication was obviously a pathogenic copy number gain; however, even so we performed array CGH analysis to obtain more detailed information. Array CGH showed an interstitial duplication on 16p13.1p13.3 with a size of about 13 Mb (arr 16p13.11-p13.3 (2,171,514-15,048,781)x3) including 98 genes.

**Fig. 2.**
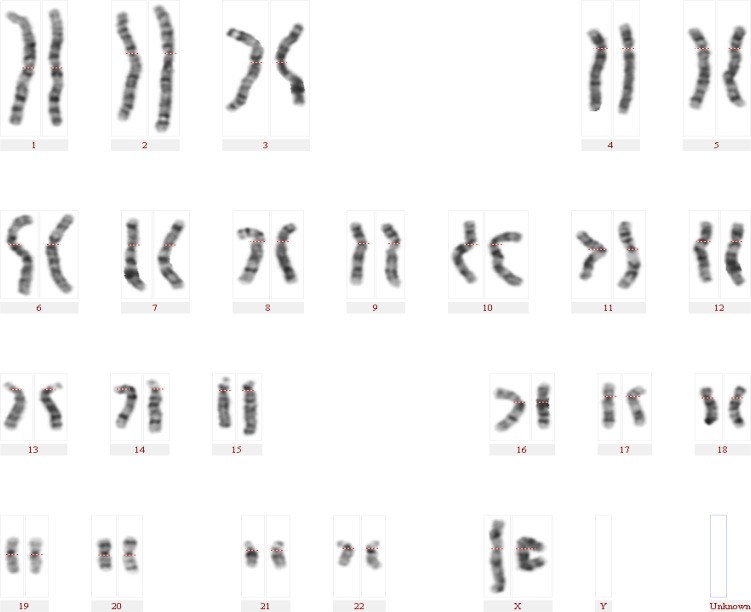
The patient (p21) karyotype: 46,XX,del(15)(q11.2)

Patient two (p-21) was an 11-year-old girl with autism, ID, microcephaly and seizure and no clinical diagnosis. In cytogenetic study, she showed a deletion on chromosome 15q deleting the nearest cytoband to the centromere (Figure 2). Her karyotype was reported as 46, XX, del(15)(q11.2). MLPA subtelomere kits confirmed the deletion in 15q11.2. The autism P343 kit demonstrated a deletion corresponding to 18 probes in 15q11.2 to 15q13.3 region with a size of about 3 Mb. The clinical and genetic findings were in concordance with the Angelman syndrome.

**CNVs detected by MLPA**

Patient 3 (p27) was a 17-year-old boy with autism, ID, microcephaly, seizure and recurrent infections. He did not show any microscopic chromosomal abnormality. Using subtelomeric P036 kit he showed a *de novo* copy number gain on 10q subtelomeric region. Nevertheless, the P070 kit did not show the duplication. It was because the 10q subtelomeric probes in the two kits are designed in 2 different genes situated next to each other in 10q26.3 region: the *ECHS1* gene, located in the centromeric side and the *PAOX* gene, located in the telomeric side, in P070 and P036 kits, respectively. The MLPA results show that the breakpoint should be somewhere between these 2 genes. The duplication size is about 200 Kb containing 8 genes: *SYC1*, *CYP2E1*, *OR7M1p*, *OR6L2P*, *SPRN*, *MTG1*, *OR6L1P* and *PAOX*. Array CGH was performed to confirm this finding and a copy number gain with a size of 237 Kb including all mentioned genes was demonstra-ted on chromosome 10q26.3. Any other clinically significant CNV was not observed using array CGH.

Patient 4 (p32) was a 13-year-old boy with autism, ID and gastrointestinal problem. Subtelomeric MLPA kits showed a copy number gain on chromosome Xp22.33 which was inherited from his normal father. In both kits the probe was located on the *SHOX* (short stature homeobox) gene.

Both patients 5 (p35) and 6 (p36) whose clinical manifestations are shown in Table 3, demonstrated normal karyotypes. Patient 5 showed a copy number gain on chromosome 15q13.3 inherited from his normal father using the autism P343 kit. Patient 6 showed a subtelomeric gain on chromosome 7q36.3 using both P036 and P070 MLPA kits. The CNV was inherited from his father. The patient’s father was examined by the neurologist and presented mild autism.

## Discussion

ASD is a clinically and etiologically heterogeneous disorder making its diagnosis difficult in the patients. Nonetheless, CNVs including microscopic and submicroscopic chromosome imbalances have been shown to be major contributors in children with autism predominantly in patients who have additional clinical features (9, 10, 12-14). Different methods are being used for CNV detection including cytogenetic, MLPA and array CGH. In this study, our aim was to investigate CNV detection rate using cytogenetic and MLPA techniques and evaluate the diagnostic yield of these two tests in patients with ASD. We selected 50 sporadic Iranian patients with autism who had ID and at least one additional clinical feature. Out of the 50 patients, 2 (4%) showed microscopic chromosome abnorma-lity in concordance with known syndromes and 5 (10%) demonstrated submicroscopic copy number gains or losses. As one of the cytogenetic and MLPA findings overlapped, our total detection rate was 6/50 (12%). The pathogenicity of the cyto-genetic findings was clear but some of MLPA results required to be better refined using more accurate techniques to decide about the patho-genecity of CNVs. The status of the pathogenicity of the CNVs identified in our patients is described as follows.

***16p13.1p13.3 duplication***

Previous studies described a characteristic phenotype for all patients with 16p13.1p13.3 duplications regardless of the size or location of the duplicated regions. The clinical features included ID, facial anomalies, fingers hypoplasia and vascular anomalies (27). Regardless, according to the more recent studies, 16p13 duplications could have been divided into different CNVs causing clinically distinguishable phenotypes: 16p13.1, 16p13.2 and 16p13.3 duplications (28). Patients who carry 16p13.11 duplications (known to be a risk factor for a wide spectrum of neurodeve-lopmetal disorders) show some recurrent clinical features including intellectual disability, autism, seizure, dysmorphic feature or congenital anomalies including microcephaly, macrocephaly and heart defects (28-31). Duplications of 16p13.2 could also be associated with ASD (32). 16p13.3 duplications encompassing the Rubinstein–Taybi region can cause another recognizable syndrome (33). The patients with such microduplication display variable phenotypes with some frequently observed clinical features such as developmental delay and intellectual disability, facial features (low frontal hair line, upslanting and short palpebral fissures, broad/depressed nasal bridge, long philtrum, low set ears) and hand and foot anomalies. In some cases also, microcephaly and kidney anomaly (hypoplastic and ectopic left kidney) were reported (27, 33-35). Besides, kidney anomaly was reported in 2 patients by Digilio et al. One of them with a duplication of 12 Mb on 16p13.3p13.13 showed an ectopic left kidney and the other with a copy number gain of about 8.5 Mb on 16p13.3p13.2 demonstrated a hypoplastic left kidney. Our patient with 16p13.1p13.3 through karyotyping showed autism, ID, microcephaly and hypoplastic kidney. In comparison, some clinical features (ID and microcephaly) can be explained by each of 16p13.1 or 16p13.3 duplications. Yet, the kidney anomaly seems to be the consequence of the copy number gain of chromosome 16p13.3 and autism should be the result of 16p13.1 duplication. 16p13.2 duplication also shows association with autism and may contribute to this phenotype.

***10q26.3 duplication***

10q distal trisomy is a rare syndrome with a variant phenotype including ID, microcephaly, facial feature, psychomotor delay, autism, recurrent infections and cardiac, renal and skeletal anomalies. The range and severity of symptoms depends on the size of the duplicated region. In different reported cases, proximal breakpoints ranged from 10q22.3 to 10q26.3 (36-39). Still, in comparison, the size of duplication in our patient is too small. The patient p27 who presented with autism, ID, microcephaly, seizure and recurrent infections showed a copy number gain with a size of about 240 Kb using both MLPA and array CGH tests. There are 8 genes in this region: *SYC1*, *CYP2E1*, *OR7M1p*, *OR6L2P*, *SPRN*, *MTG1*, *OR6L1P* and *PAOX*. According to UCSC and SFARI databases, some of the mentioned genes including *PAOX*, *MTG1* and *SYCE1* have an expression and function in brain, from which *SYCE1* has been also reported as a risk factor for autism (40). Notwithstanding, there is contradictory information on databases and literature. There are similar duplications in patients with autism reported in DECIPHER database. In addition, there is a report of a patient with autism, ID and microcephaly who showed duplication with a size of 170 Kb containing *PAOX*, *MTG1* and *SPRN* genes (41). On the other hand, this copy number gain has been reported several times in DGV in normal population. Therefore this copy number gain may have no clinical significance or have pathogenic impact with an incomplete penetrance. Because of these incompatible data and lack of evidence for dosage-sensitivity of the included genes, we consider this duplication as a clinically uncertain CNV. Future studies in autism patients may elucidate the pathogenecity statement of this CNV.

***Xp22.33 duplication***

Different studies have shown that many genomic rearrangements occur in the subtelomeric region of Xp which can cause various disorders including autism (15, 42, 43). In spite of that, the copy number gain in our patient (p32) has been inherited from his normal father and it seems to be a benign CNV. Thus the autism and other clinical features in patient p32 are probably caused by the other copy number or single nucleotide variation(s) which need more accurate and whole genome techniques to be identified.

***15q13.3 and 7qsubtel duplications***

15q13.3 duplication was observed in patient p35. His normal father also demonstrated the same CNV. Duplications and deletions of this region are known as recurrent CNVs involved in ASD with an incomplete penetrance which may be passed on to the affected patients from normal parents (44). Hence, further studies are needed to determine the size and gene content of the CNV to decide about its pathogenicity status and detect the other possible genetic variant(s) in the patient’s genome. Such a variant could be a copy number and/or a single nucleotide variation with a role in ASD phenotype as a second hit. Patient p36 showed a subtelomeric gain on chromosome 7q36.3 using both P036 and P070 MLPA kits, inherited from his father. The patient’s father was examined by the neurologist and was diagnosed as having mild autism. 7q36.3 duplication has been proposed to be involved in autism etiology (45). Therefore, there is possibility for this CNV to be involved in autism phenotype in both son and father. However, MLPA cannot give information about the size of duplication and its gene content. Hence, it is necessary to use a more accurate technique such as array CGH to determine the gene content of this region.

## Conclusions

Cytogenetic study defined the genetic causes in 2 (4%) patients, both of them were pathogenic CNVs. MLPA test demonstrated copy number changes in 5 (10%) patients, 4 (8%) of them were detected by subtelomeric kits and 2 (4%) others were identified by the autism P343 kit (there was one overlapping detection). The P393 kit (*SHANK2* gene) did not identify any abnormality. Our findings suggest that subtelomeric MLPA kits that are recommended for chromosome analysis in patients with ID (with a detection rate of about 6%) can give some beneficial data in autistic patients especially when they show additional features including ID. We expect that the autism kits with the probes covering the most recurrent CNVs in autism, 15q11-q13 and 16p11 regions together with *SHANK3* and *SHANK2 *genes, have a detection rate of about 2-6%. The detection rate using these kits was 1/50 (2%).

All detected CNVs but one were copy number gains. Out of 6 CNVs, three were *de novo* and three other were paternally inherited. One of inherited CNVs seems to be a benign CNV (Xp22.33 duplication) and the other (15q13.3 duplication) may have an incomplete penetrance. But the third CNV was 7q36.3 duplication which was inherited from the patient’s father. After further examination he showed mild autism. As a consequence, this CNV may have pathogenic impact in both son and father.
